# Outcomes beyond the Third Month of Anticoagulation in Patients Aged >75 Years with a First Episode of Unprovoked Venous Thromboembolism

**DOI:** 10.1055/s-0038-1676359

**Published:** 2018-12-10

**Authors:** Amaia Iñurrieta, José María Pedrajas, Manuel Jesús Núñez, Luciano López-Jiménez, Alba Velo-García, Juan Carlos García, Ramón Lecumberri, David Jiménez, Isaac Pons, Manuel Monreal, the RIETE Investigators

**Affiliations:** 1 Department of Internal Medicine, Hospital Clínico San Carlos, Madrid, Spain; 2 Department of Internal Medicine, Complejo Hospitalario de Pontevedra, Pontevedra, Spain; 3 Department of Internal Medicine, Hospital Universitario Reina Sofía, Córdoba, Spain; 4 Department of Haematology, Clínica Universidad de Navarra, Pamplona, Spain; 5 Department of Pneumonology, Hospital Ramón y Cajal, Madrid, Spain; 6 Department of Internal Medicine, Hospital de Igualada, Barcelona, Spain; 7 Department of Internal Medicine, Universidad Católica de Murcia, Hospital de Badalona Germans Trias i Pujol, Murcia, Spain

**Keywords:** venous thromboembolism, age, anticoagulant therapy, hemorrhage, mortality

## Abstract

**Background** The ideal duration of anticoagulant therapy in elderly patients with unprovoked venous thromboembolism (VTE) has not been consistently evaluated.

**Methods** We used the RIETE ( *R* egistro *I* nformatizado *E* nfermedad *T* rombo *E* mbólica) registry to compare the rate and severity of pulmonary embolism (PE) recurrences versus major bleeding beyond the third month of anticoagulation in patients >75 years with a first episode of unprovoked VTE.

**Results** As of September 2017, 7,830 patients were recruited: 5,058 (65%) presented with PE and 2,772 with proximal deep vein thrombosis (DVT). During anticoagulant therapy beyond the third month (median, 113 days), 44 patients developed PE recurrences, 36 developed DVT recurrences, 101 had major bleeding, and 241 died (3 died of recurrent PE and 19 of bleeding). The rate of major bleeding was twofold higher than the rate of PE recurrences (2.05 [95% confidence interval, CI: 1.68–2.48] vs. 0.90 [95% CI: 0.66–1.19] events per 100 patient-years) and the rate of fatal bleeding exceeded the rate of fatal PE events (0.38 [95% CI: 0.24–0.58] vs. 0.06 [95% CI: 0.02–0.16] deaths per 100 patient-years). On multivariable analysis, patients who had bled during the first 3 months (hazard ratio [HR]: 4.32; 95% CI: 1.58–11.8) or with anemia at baseline (HR: 1.87; 95% CI: 1.24–2.81) were at increased risk for bleeding beyond the third month. Patients initially presenting with PE were at increased risk for PE recurrences (HR: 3.60; 95% CI: 1.28–10.1).

**Conclusion** Prolonging anticoagulation beyond the third month was associated with more bleeds than PE recurrences. Prior bleeding, anemia, and initial VTE presentation may help decide when to stop therapy.

## Introduction

The decision to prolong or to discontinue anticoagulant therapy beyond the third month in elderly patients with unprovoked venous thromboembolism (VTE) has not been consistently addressed so far. Current guidelines of antithrombotic therapy, based on the results of randomized trials, recommend that patients with unprovoked VTE be treated with anticoagulants for at least 3 months (Grade 1B). 1 Then, they suggest to prolong anticoagulation in patients at low or moderate risk for bleeding (Grade 2B) and recommend its discontinuation in those with a high risk for bleeding (Grade 1B). Both the frequency of unprovoked VTE and the risk of bleeding progressively increase with age, 2 3 4 5 6 7 but elderly patients are often excluded from randomized clinical trials, and the ideal duration of anticoagulation in this patient population remains unknown.

The RIETE ( *R* egistro *I* nformatizado de *E* nfermedad *T* rombo *E* mbólica) registry is an ongoing, multicenter, international observational registry of consecutive patients with objectively confirmed, acute VTE (ClinicalTrials.gov identifier: NCT02832245). It started in Spain in 2001, and 6 years later the database was translated into English with the aim to expand the registry to other countries, ultimately allowing physicians worldwide to use the database to select the most appropriate therapy for their patients. Data from this registry have been used to evaluate outcomes after acute VTE, such as the frequency of recurrent VTE, bleeding and mortality, and risk factors for these outcomes. 8 9 10 11 12 13 The current study analyzed the rate and severity of VTE recurrences and major bleeding events occurring beyond the third month of anticoagulation in patients aged >75 years with a first episode of unprovoked VTE. Then, we tried to identify independent predictors for recurrent pulmonary embolism (PE) and for major bleeding.

## Patients and Methods

### Inclusion Criteria

Consecutive patients with symptomatic, acute deep vein thrombosis (DVT) or PE confirmed by objective tests (compression ultrasonography or contrast venography for DVT; helical CT-scan, ventilation-perfusion lung scintigraphy, or angiography for PE) were enrolled in RIETE. The rationale and methodology of RIETE has been recently published. 14 Patients were excluded if they were currently participating in a therapeutic clinical trial with a blinded therapy. All patients (or their relatives) provided written or oral consent for participation in the registry, in accordance with local ethics committee requirements.

### Study Design

For this study, only patients aged >75 years with a first episode of unprovoked PE or proximal DVT were considered. VTE was considered to be unprovoked in the absence of active cancer, recent (<2 months before) immobility, surgery, bone fracture, or long-term travel. DVT was considered proximal if it is located above the popliteal vein. The major outcome was the rate of symptomatic PE recurrences and major bleeding events occurring beyond the first 3 months of anticoagulant therapy. Secondary outcomes were fatal PE recurrences and fatal bleeding. Major bleeding was defined as an overt bleed requiring a transfusion of two or more units of blood, or if it was retroperitoneal, spinal, intracranial, or fatal. Fatal bleeding was defined as any death occurring within 10 days of a major bleeding episode, in the absence of an alternative cause of death. Fatal recurrent PE, in the absence of autopsy, was defined as any death appearing within 10 days of a symptomatic, objectively confirmed PE recurrence, in the absence of any alternative cause of death.

### Baseline Variables

The following parameters were recorded when the qualifying episode of VTE was diagnosed: patient's gender, age and body weight, presence of coexisting conditions, risk factors, concomitant diseases and medications, laboratory data at baseline, and treatment details (drug, dose, and duration). Unfortunately, there is information only on the clinical characteristics of patients or laboratory tests at baseline (not on day 90).

### Treatment and Follow-up

Patients were managed according to the clinical practice of each participating hospital (i.e., there was no standardization of therapy). The drug, dose, and duration of anticoagulant therapy were recorded. The decision on the type and duration of therapy was left to the attending physicians. Patients were followed up in the outpatient clinic (or telephone interviews for patients who could not show up for a clinic visit). During each visit, any signs or symptoms suggesting VTE recurrences or major bleeding were noted. Each episode of clinically suspected recurrent VTE was investigated by repeat compression ultrasonography, lung scan, helical-CT scan, or pulmonary angiography, as appropriate. Most outcomes were classified as reported by the clinical centers.

### Statistical Analysis

Analysis of variance and nonparametric tests were used to compare means and medians of continuous variables. Categorical variables were compared using the chi-square test (two-sided) and Fisher's exact test (two-sided). Incidence rates of recurrent VTE, major bleeding, and death were calculated as cumulative incidence (events/100 patient-years). To measure predictors of outcome, a multivariate analysis was performed using a Cox proportional hazard regression analyses, and the corresponding Kaplan–Meier survival curves were built. Candidate variables were selected from clinical variables based on published literature and on expert opinion. Cut-offs for age and body weight were chosen arbitrarily. Crude and adjusted hazard ratios (HRs) as well as their 95% confidence intervals (CIs) were estimated. Covariates included in the adjusted model were those for which a statistically significant difference (a threshold *p* -value of 0.1 was set to assess significance of differences) was found, and a backward selection was used for the covariate selection in the multivariable model. SPSS software (version 20, SPSS Inc., Chicago, Illinois, United States) was used for the statistical management of the data, and a two-sided *p* < 0.05 was considered to be statistically significant.

## Results

As of September 2017, 7,830 patients aged >75 years with a first episode of unprovoked VTE were still alive beyond the third month of anticoagulant therapy. Of these, 5,058 patients (65%) had initially presented with PE (with or without concomitant DVT) and 2,772 with proximal DVT alone. Patients initially presenting with PE were more likely to be women and to have arterial hypertension, chronic heart failure, chronic lung disease, prior artery disease, or renal insufficiency, and were less likely to have anemia than those initially with DVT ( Table 1 ). In addition, patients initially presenting with PE were more likely to be using nonsteroidal anti-inflammatory drugs or antiplatelets at baseline than those presenting with DVT alone. Most patients in both subgroups (89 and 95%, respectively) received initial therapy with low-molecular-weight heparin (LMWH). Then, most (81 and 74%) switched to vitamin K antagonists (VKAs). Mean duration of therapy beyond the third month was longer in PE patients than in those with DVT ( Table 2 ). One in every three patients (62%) was still receiving anticoagulant therapy 180 days after the index event, and one in every four patients (26%) was still receiving anticoagulant therapy 1 year later.

**Table 1:** Clinical characteristics at baseline and treatment, according to initial VTE presentation

	Pulmonary embolism	Proximal deep vein thrombosis	Odds ratio (95% CI)
Patients, *N*	5,058	2,772	
Clinical characteristics			
Gender (male)	1,795 (35%)	1,198 (43%)	0.72 (0.66–0.79)
Age (y ± SD)	82 ± 4.7	82 ± 4.7	–
Weight (kg ± SD)	72 ± 13	72 ± 13	–
Concomitant diseases			
Diabetes mellitus	594 (12%)	298 (11%)	1.10 (0.95–1.28)
Arterial hypertension	2,377 (47%)	1,058 (38%)	1.44 (1.31–1.58)
Chronic heart failure	718 (14%)	180 (6.5%)	2.38 (2.01–2.83)
Chronic lung disease	818 (16%)	291 (10%)	1.64 (1.43–1.90)
Prior myocardial infarction	378 (7.5%)	132 (4.8%)	1.62 (1.32–1.98)
Prior ischemic stroke	350 (6.9%)	111 (4.0%)	1.78 (1.43–2.22)
Recent major bleeding	40 (0.79%)	13 (0.47%)	1.69 (0.90–3.17)
Major bleeding within 3 mo	56 (1.1%)	26 (0.94%)	1.18 (0.74–1.89)
Events from day 0 to day 90			
Major bleeding	56 (1.1%)	26 (0.94%)	1.18 (0.74–1.89)
PE recurrences	11 (0.22%)	7 (0.25%)	0.86 (0.33–2.22)
Concomitant therapies			
Corticosteroids	350 (6.9%)	167 (6.0%)	1.16 (0.96–1.40)
NSAIDs	287 (5.7%)	124 (4.5%)	1.28 (1.04–1.59)
Antiplatelets	1,246 (25%)	472 (17%)	1.59 (1.42–1.79)
Laboratory tests at baseline			
CrCl levels < 30 mL/min	631 (12%)	312 (11%)	1.12 (0.97–1.30)
CrCl levels < 60 mL/min	3,704 (73%)	1,916 (69%)	1.22 (1.10–1.35)
Anemia	1,312 (26%)	868 (31%)	0.77 (0.69–0.85)
Abnormal platelet count	170 (3.4%)	86 (3.1%)	1.09 (0.83–1.41)
Initial therapy			
Low-molecular-weight heparin	4,502 (89%)	2,642 (95%)	0.40 (0.33–0.49)
Unfractionated heparin	372 (7.4%)	50 (1.8%)	4.32 (3.20–5.83)
Direct oral anticoagulants	33 (0.65%)	39 (1.4%)	0.46 (0.29–0.73)
Fondaparinux	58 (1.1%)	26 (0.94%)	1.23 (0.77–1.95)
Vena cava filter	49 (0.97%)	10 (0.36%)	2.70 (1.37–5.34)
Long-term therapy			
Vitamin K antagonists	4,077 (81%)	2,053 (74%)	1.46 (1.30–1.62)
Low-molecular-weight heparin	705 (14%)	563 (20%)	0.64 (0.56–0.72)
Direct oral anticoagulants	246 (4.9%)	130 (4.7%)	1.04 (0.84–1.29)
Duration of therapy			
Mean days ± SD	261 ± 395	181 ± 285	–
Median days (IQR)	125 (57–288)	99 (33–210)	–
Patients treated > 6 mo	3,308 (65%)	1,574 (57%)	1.44 (1.31–1.58)
Patients treated > 12 mo	1,500 (30%)	533 (19%)	1.77 (1.58–1.98)

Abbreviations: CI, confidence intervals; CrCl, creatinine clearance; IQR, interquartile range; NSAIDs, nonsteroidal anti-inflammatory drugs; PE, pulmonary embolism; SD, standard deviation; VTE, venous thromboembolism.

**Table 2:** Clinical outcomes beyond the third month of anticoagulant therapy, according to initial VTE presentation and treatment

	All patients		Pulmonary embolism		Proximal DVT	
	*N*	Events per 100 patient-years	*N*	Events per 100 patient-years	*N*	Events per 100 patient-years
Patients, *N*	7,830		5,058		2,772	
Duration of therapy						
Mean days ± SD	232 ± 362		261 ± 395		181 ± 285***	
Median days (IQR)	113 (46–275)		125 (57–288)		99 (33–210)***	
Recurrent PE	44	0.90 (0.66–1.19)	40	1.13 (0.82–1.52)	4	0.29 (0.09–0.70)**
Recurrent DVT	36	0.73 (0.52–1.00)	14	0.39 (0.22–0.64)	22	1.63 (1.05–2.43)***
Major bleeding	101	2.05 (1.68–2.48)	71	1.98 (1.56–2.49)	30	2.21 (1.52–3.12)
Sites of major bleeding						
Gastrointestinal	40	0.81 (0.58–1.09)	23	0.64 (0.41–0.94)	17	1.25 (0.75–1.96)*
Cerebral	37	0.75 (0.53–1.02)	31	0.86 (0.60–1.21)	6	0.44 (0.18–0.91)
Death	241	4.84 (4.26–5.48)	198	5.49 (4.76–6.29)	43	3.13 (2.30–4.18)***
Causes of death						
Pulmonary embolism	3	0.06 (0.02–0.16)	2	0.06 (0.01–0.18)	1	0.07 (0.00–0.36)
Bleeding	19	0.38 (0.24–0.58)	17	0.47 (0.28–0.74)	2	0.15 (0.02–0.48)
Patients on VKA, *N*	6,130		4,077		2,053	
Recurrent PE	31	0.75 (0.52–1.06)	27	0.91 (0.61–1.30)	4	0.35 (0.11–0.85)
Recurrent DVT	31	0.75 (0.52–1.05)	12	0.40 (0.22–0.68)	19	1.70 (1.05–2.60)***
Major bleeding	81	1.97 (1.57–2.43)	57	1.91 (1.46–2.45)	24	2.14 (1.40–3.13)
Death	183	4.40 (3.80–5.07)	157	5.20 (4.43–6.06)	26	2.28 (1.52–3.30)***
Patients on LMWH, *N*	1,268		705		563	
Recurrent PE	11	1.83 (0.96–3.17)	11	2.59 (1.36–4.51)	0	–
Recurrent DVT	4	0.65 (0.21–1.57)	2	0.45 (0.08–1.50)	2	1.15 (0.19–3.79)
Major bleeding	17	2.74 (1.65–4.30)	11	2.48 (1.31–4.32)	6	3.39 (1.37–7.04)
Death	51	8.20 (6.17–10.7)	38	8.57 (6.15–11.6)	13	7.29 (4.06–12.2)
Patients on DOACs, *N*	376		246		130	
Recurrent PE	2	1.13 (0.19–3.75)	2	1.56 (0.26–5.16)	0	–
Recurrent DVT	1	0.57 (0.03–2.79)	0	–	1	2.10 (0.11–10.4)
Major bleeding	2	1.13 (0.19–3.72)	2	1.55 (0.26–5.12)	0	–
Death	4	2.25 (0.72–5.44)	2	1.55 (0.26–5.12)	2	4.14 (0.69–13.7)

Abbreviations: CI, confidence intervals; DOACs, direct oral anticoagulants; DVT, deep vein thrombosis; IQR, interquartile range; LMWH, low-molecular-weight heparin; PE, pulmonary embolism; SD, standard deviation; VKA, vitamin K antagonists.

Note: Comparisons between patients with PE or DVT: * *p* < 0.05; ** *p* < 0.01; *** *p* < 0.001.

**Fig. 1:**
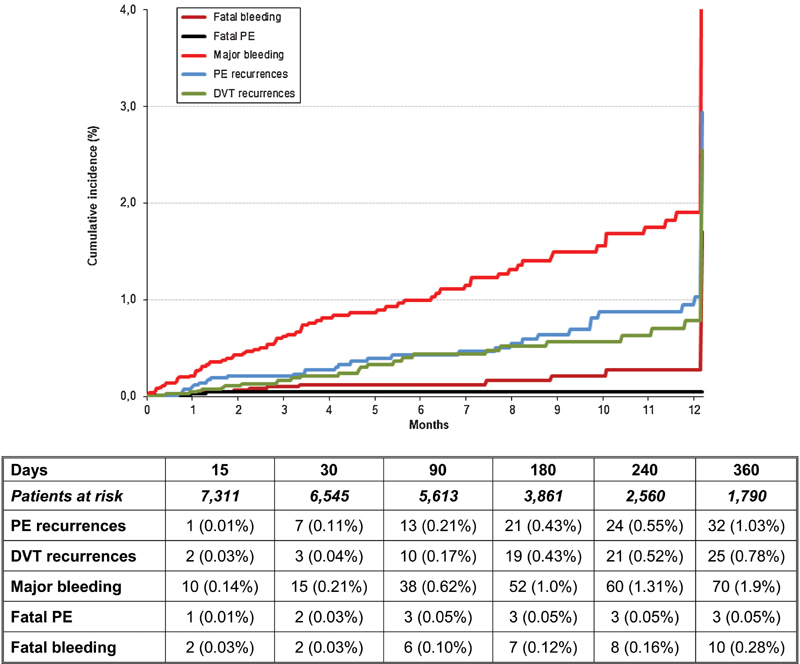
Cumulative rates of recurrent PE, recurrent DVT, major bleeding, fatal PE, and fatal bleeding during anticoagulant therapy (beyond the third month). Day 0 in the figure means day 91 of anticoagulant therapy. DVT, deep vein thrombosis; PE, pulmonary embolism.

During the course of anticoagulation beyond the third month (median, 113 days), 44 patients developed PE recurrences, 36 developed DVT recurrences, 101 had major bleeding, and 241 died (3 died of recurrent PE and 19 of bleeding). The rate of major bleeding was twofold higher than the rate of PE recurrences (2.05 [95% CI: 1.68–2.48] vs. 0.90 [95% CI: 0.66–1.19] events per 100 patient-years) and the rate of fatal bleeding far exceeded the rate of fatal PE events (0.38 [95% CI: 0.24–0.58] vs. 0.06 [95% CI: 0.02–0.16] deaths per 100 patient-years) ( Fig. 1 ). Among patients initially presenting with PE, 40 patients developed PE recurrences, 14 had DVT recurrences, 71 had major bleeding, and 198 died (2 died of recurrent PE and 17 of bleeding). Among those initially presenting with DVT, there were 4 PE recurrences, 22 DVT recurrences, 30 major bleeds, and 43 deaths (1 patient died of recurrent PE, 2 died of bleeding). Thus, in patients initially presenting with PE, the rate of PE recurrences was higher than in those with DVT alone (1.13 [95% CI: 0.82–1.52] vs. 0.29 [95% CI: 0.09–0.70] events per 100 patient-years; *p* < 0.01), but the rate of DVT recurrences was lower (0.39 [95% CI: 0.22–0.64] vs. 1.63 [95% CI: 1.05–2.43] events per 100 patient-years; *p* < 0.001), as shown in Table 2 . There were no differences in the rate of major bleeding, but patients initially presenting with PE had a lower rate of gastrointestinal bleeding than those initially presenting with DVT. When separately analyzing outcomes according to prescribed drugs, the rate of major bleeding exceeded the rate of PE recurrences during the course of therapy in patients receiving VKAs (81 vs. 31 events, respectively) and in those on LMWH (17 vs. 11 events, respectively), as also shown in Table 2 . The amount of patients receiving direct oral anticoagulants was too small to make comparisons.

On multivariable analysis, after adjusting for patient's age, sex, body weight, initial VTE presentation (PE vs. DVT alone), underlying diseases, concomitant therapies, renal function, anemia, thrombocytopenia, presence of PE recurrences or major bleeding during the first 3 months, and type of long-term therapy, patients with major bleeding during the first 3 months of therapy (HR: 4.32; 95% CI: 1.58–11.8) and those presenting with anemia at baseline (HR: 1.87; 95% CI: 1.24–2.81) were at increased risk for major bleeding beyond the third month ( Table 3 ). Moreover, initial presentation as PE at baseline was the only variable that independently predicted risk for PE recurrences (HR: 3.60; 95% CI: 1.28–10.1).

**Table 3:** Uni- and multivariable analysis for major bleeding and for PE recurrences

	Major bleeding		PE recurrences	
	Nonadjusted HR (95% CI)	Adjusted HR (95% CI)	Nonadjusted HR (95% CI)	Adjusted HR (95% CI)
Patients, *N*	101		44	
Clinical characteristics				
Gender (male)	1.33 (0.89–1.96)	–	0.65 (0.34–1.25)	–
Age > 82 y	1.50 (1.01–2.22)	1.30 (0.86–1.96)	1.69 (0.93–3.05)	1.38 (0.74–2.58)
Weight > 70 kg	0.73 (0.49–1.08)	–	0.52 (0.28–0.96)	0.58 (0.30–1.13)
Initial VTE presentation				
Pulmonary embolism	0.92 (0.60–1.41)	0.91 (0.59–1.41)	3.99 (1.42–11.16)	3.60 (1.28–10.1)
Concomitant diseases				
Diabetes	1.09 (0.62–1.93)	–	1.03 (0.44–2.45)	–
Arterial hypertension	1.45 (0.98–2.16)	1.38 (0.92–2.08)	1.11 (0.62–2.01)	–
Chronic heart failure	0.78 (0.41–1.50)	–	2.37 (1.20–4.69)	1.86 (0.93–3.73)
Chronic lung disease	1.11 (0.65–1.89)	–	0.74 (0.29–1.87)	–
Prior myocardial infarction	1.10 (0.53–2.26)	–	1.98 (0.84–4.69)	–
Prior ischemic stroke	1.71 (0.91–3.19)	1.49 (0.79–2.82)	2.13 (0.90–5.05)	1.67 (0.70–3.99)
Recent major bleeding	1.92 (0.27–13.8)	–	4.66 (0.64–33.9)	–
Events from day 0 to day 90				
Major bleeding	4.40 (1.62–12.0)	4.32 (1.58–11.8)	2.38 (0.33–17.3)	–
PE recurrences	–	–	–	–
Concomitant therapies				
Corticosteroids	1.58 (0.82–3.04)	–	1.02 (0.32–3.30)	–
NSAIDs	0.84 (0.34–2.08)	–	2.07 (0.82–5.25)	–
Antiplatelets	0.86 (0.53–1.40)	–	1.66 (0.89–3.09)	–
Laboratory tests at baseline				
CrCl levels > 60 mL/min	(Ref.)	(Ref.)	(Ref.)	(Ref.)
CrCl levels 30–60 mL/min	1.20 (0.74–1.92)	1.08 (0.67–1.76)	1.22 (0.59–2.54)	0.85 (0.39–1.87)
CrCl levels < 30 mL/min	1.87 (1.01–3.49)	1.37 (0.72–2.63)	2.40 (0.98–5.91)	1.23 (0.45–3.37)
Anemia	2.01 (1.35–3.01)	1.87 (1.24–2.81)	0.91 (0.45–1.85)	–
Abnormal platelet count	0.05 (0.00–6.15)	–	0.67 (0.09–4.84)	–
Long-term therapy				
Vitamin K antagonists	0.82 (0.50–1.35)	–	0.48 (0.25–0.92)	0.81 (0.19–3.43)
LMWH	1.38 (0.82–2.33)	–	2.33 (1.18–4.61)	1.77 (0.39–8.10)
Direct oral anticoagulants	0.53 (0.13–2.15)	–	1.23 (0.30–5.08)	–

Abbreviations: CI, confidence intervals; CrCl, creatinine clearance; HR, hazard ratio; LMWH, low-molecular-weight heparin; NSAIDs, nonsteroidal anti-inflammatory drugs; PE, pulmonary embolism; Ref, reference; VTE, venous thromboembolism.

After excluding 82 patients who developed major bleeding during the first 3 months of therapy, the rate of major bleeding outweighed the rate of PE recurrences in patients initially presenting with DVT (28 vs. 4 events, respectively) and in those presenting with PE and anemia (23 vs. 8 events, respectively), as shown in Table 4 . In PE patients without anemia, however, both rates were closer (46 vs. 31 events).

**Table 4:** Clinical outcomes during anticoagulation in the 7,748 patients that did not bled during the first 3 months, according to initial VTE presentation and the presence or absence of anemia at baseline

	Anemia		No anemia		Hazard ratio (95% CI)
	*N*	Events per 100 patient-years	*N*	Events per 100 patient-years	
PE patients, *N*	1,291		3,711		
Recurrent DVT	2	0.26 (0.04–0.87)	12	0.43 (0.23–0.73)	0.61 (0.09–2.43)
Recurrent PE	8	1.07 (0.50–2.03)	31	1.12 (0.78–1.58)	0.95 (0.41–2.00)
Major bleeding	23	3.04 (1.97–4.49)	46	1.65 (1.22–2.18)	1.84 (1.10–3.02)
Sites of major bleeding					
Intracranial	7	0.92 (0.40–1.82)	22	0.79 (0.51–1.17)	1.17 (0.46–2.67)
Gastrointestinal	10	1.32 (0.67–2.35)	13	0.46 (0.26–0.77)	2.85 (1.21–6.55)
Death	68	8.95 (7.00–11.3)	127	4.52 (3.78–5.36)	1.98 (1.47–2.65)
DVT patients, *N*	856		1,890		
Recurrent DVT	7	1.74 (0.76–3.44)	15	1.60 (0.93–2.58)	1.09 (0.41–2.63)
Recurrent PE	2	0.49 (0.08–1.60)	2	0.21 (0.04–0.70)	2.30 (0.24–22.1)
Major bleeding	14	3.49 (1.99–5.72)	14	1.48 (0.84–2.42)	2.36 (1.11–5.03)
Sites of major bleeding					
Gastrointestinal	10	2.48 (1.26–4.42)	6	0.63 (0.26–1.32)	3.92 (1.42–11.6)
Intracranial	1	0.24 (0.01–1.20)	5	0.53 (0.19–1.17)	0.46 (0.02–3.33)
Death	14	3.39 (1.93–5.56)	29	3.05 (2.08–4.33)	1.11 (0.57–2.09)

Abbreviations: CI, confidence intervals; DVT, deep vein thrombosis; PE, pulmonary embolism.

## Discussion

Our findings, obtained from a large series of patients aged >75 years with a first episode of unprovoked VTE, reveal that the rate of major bleeding beyond the third month of therapy was over twofold higher than the rate of PE recurrences (101 vs. 44 events, respectively), and the rate of fatal bleeding also outweighed the rate of fatal PE (19 vs. 3 deaths). The higher rate and severity of major bleeding than recurrent PE was consistently found in patients receiving long-term therapy with VKAs or LMWH. Therefore, the clinical relevance of bleeding beyond the third month should not be underestimated, even in patients that did not bleed during the first 3 months. On the contrary, accurate identification of at-risk patients is urgently needed.

In our cohort, patients presenting with major bleeding during the first 3 months had an over fourfold higher risk to rebleed beyond the third month. We firmly believe that most of these patients would have benefited from earlier discontinuation of anticoagulant therapy. This is the reason why we re-evaluated the outcomes after excluding patients that bled during the first 3 months. Then, we found that patients initially presenting with DVT (2,746 of 7,748 patients, 35%) had a particularly low rate of PE recurrences, and those with anemia at baseline (28%) had a much higher rate of major bleeding than those without anemia. The higher risk for bleeding in patients with anemia has already been reported previously. 1 2 4 5 15 16 17 18 The lower rate of PE recurrences in patients initially presenting with DVT than in those with PE has also been reported in several studies. 19 20 21 Hence, adequately designed, randomized trials comparing only 3 months versus prolonged anticoagulation beyond the third month in patients with DVT or anemia would likely provide information about the ideal duration of therapy in this patient population. In the meantime, we suggest that most patients aged >75 years presenting with unprovoked DVT or with anemia at baseline would likely benefit from early (3 months) discontinuation of therapy.

The present study has several limitations. First, RIETE is an observational registry, not a randomized trial. Our data are hypothesis-generating and might be a useful basis for future controlled clinical trials, so we should be extremely cautious in suggesting changes in treatment strategies just because of registry data. Second, treatment varied with local practice, and is likely to have been influenced by a physician's assessment of patient's risk of bleeding. Third, it is mandatory in RIETE that patients must experience an objectively confirmed recurrent PE followed by death within the previous 10 days to be considered as fatal PE. Thus, it is possible that some deaths of unknown cause were truly PE-related deaths. However, some patients may have also died of myocardial infarction, ischemic stroke, or even intracranial bleeding. Finally, the numbers of patients in the respective subgroups tend to be small, which may render the statistical analyses meaningless. Strengths of the current analysis include that the RIETE registry provides data on the treatment of VTE in a real-world situation with an unselected patient population, in contrast to the rigorously controlled conditions of randomized clinical trials. This is important because our patient population reflects routine, unmonitored medical practice involving a broad spectrum of patients with VTE. Moreover, all VTE recurrences were confirmed by objective tests.

In summary, prolonging anticoagulant therapy beyond the third month in patients aged >75 years with unprovoked VTE was associated with a higher rate of major bleeding than PE recurrences, and a higher rate of fatal bleeding than fatal PE. Prior bleeding during the first 3 months, anemia at baseline, and initial VTE presentation may help decide when to stop therapy.

## Notes

Appendix A
**Coordinator of the RIETE Registry:** Monreal M (Spain).

**RIETE Steering Committee Members:** Decousus H (France), Prandoni P (Italy), Brenner B (Israel).

**RIETE National Coordinators:** Barba R (Spain), Di Micco P (Italy), Bertoletti L (France), Schellong S (Germany), Tzoran I (Israel), Reis A (Portugal), Bosevski M (R. Macedonia), Bounameaux H (Switzerland), Malý R (Czech Republic), Wells P (Canada), Papadakis M (Greece).

**RIETE Registry Coordinating Center:** S & H Medical Science Service.

**Members of the RIETE Group:** SPAIN: Adarraga MD, Andújar V, Arcelus JI, Ballaz A, Barba R, Barrón M, Barrón-Andrés B, Bascuñana J, Blanco-Molina A, Bueso T, Cañas I, Casado I, del Molino F, del Toro J, Díaz JA, Falgá C, Fernández-Capitán C, Font L, Gallego P, García-Bragado F, Gómez V, González J, Grau E, Guijarro R, Guil M, Guirado L, Gutiérrez J, Hernández-Blasco L, Hernández-Huerta S, Jara-Palomares L, Jaras MJ, Jiménez D, Jiménez R, Lacruz B, Lecumberri R, Lobo JL, López-Jiménez L, López-Reyes R, López-Sáez JB, Lorente MA, Lorenzo A, Madridano O, Maestre A, Marchena PJ, Martín-Antorán JM, Martín-Martos F, Monreal M, Morales MV, Nauffal D, Nieto JA, Núñez MJ, Odriozola M, Otalora S, Otero R, Pagán B, Pedrajas JM, Peris ML, Pons I, Porras JA, Riera-Mestre A, Rivas A, Rodríguez-Dávila MA, Rosa V, Rosa-Silvia A, Ruiz-Giménez N, Ruiz-Martínez C, Sabio P, Sampériz A, Sánchez R, Sanz O, Soler S, Suriñach JM, Tiberio G, Tirado R, Tolosa C, Trujillo-Santos J, Uresandi F, Valero B, Valle R, Vela J, Vilar C, Villalobos A, Villalta J, ARGENTINA: Malfante P, BELGIUM: Vanassche T, Verhamme P, CANADA: Wells P, CZECH REPUBLIC: Hirmerova J, Malý R, Tomko T, FRANCE: Bertoletti L, Bura-Riviere A, Farge-Bancel D, Hij A, Mahe I, Merah A, Moustafa F, GERMANY: Schellong S, GREECE: Babalis D, Papadakis M, Tzinieris I, ISRAEL: Braester A, Brenner B, Tzoran I, ITALY: Apollonio A, Barillari G, Bucherini E, Camerota A, Ciammaichella M, Di Micco P, Ferrazzi P, Maida R, Mastroiacovo D, Pace F, Pasca S, Pesavento R, Pinelli M, Piovella C, Prandoni P, Rota L, Tiraferri E, Tonello D, Tufano A, Visonà A, Zalunardo B, LATVIA: Skride A, PORTUGAL: Mafalda A, Ribeiro JL, Sousa MS, REPUBLIC OF MACEDONIA: Bosevski M, SWITZERLAND: Alatri A, Bounameaux H, Calanca L, Mazzolai L, VENEZUELA: Serrano JC.
